# Haemodynamic Effects of Anaemia in Patients with Acute Decompensated Heart Failure

**DOI:** 10.1155/2020/9371967

**Published:** 2020-03-21

**Authors:** Paweł Krzesiński, Agata Galas, Grzegorz Gielerak, Beata Uziębło-Życzkowska

**Affiliations:** Department of Cardiology and Internal Diseases, Military Institute of Medicine, Szaserów Street 128, 04-141 Warsaw, Poland

## Abstract

Anaemia is a common comorbidity in patients with heart failure (HF) and is associated with more severe symptoms and increased mortality. The aim of this study was to evaluate haemodynamic profiles of HF patients with respect to the presence of reduced left ventricular ejection fraction (LVEF) and anaemia. *Methods and Results*. Haemodynamic status was evaluated in 97 patients with acute decompensated HF. Impedance cardiography, echocardiography, and N-terminal probrain natriuretic peptide (NT-proBNP) results were analysed. The study group was stratified into four subgroups according to LVEF (<40% vs ≥40%) and the presence of anaemia (haemoglobin <13.0 g/dL in men and <12.0 g/dL in women). Thoracic fluid content was higher (*p*=0.037) in anaemic subjects, while no significant relation between anaemia and NYHA was observed. Anaemic subjects with LVEF ≥ 40% were distinguished from those with LVEF < 40% by significantly higher stroke index (*p*=0.002), Heather index (*p*=0.014), and acceleration index (*p*=0.047). Patients with reduced LVEF and anaemia presented the highest NT-proBNP (*p*=0.003). *Conclusions*. In acute decompensated HF, anaemia is related with fluid overload, relatively higher cardiac systolic performance but no clinical benefit in patients with preserved/midrange LVEF, and increased left ventricular tension, fluid overload, and impaired cardiac systolic performance in patients with reduced LVEF.

## 1. Introduction

Anaemia is a common comorbidity in patients with heart failure (HF) and is associated with increased symptom severity and higher mortality [1, 2]. Even a small reduction in haemoglobin (Hb) concentration is associated with less favourable outcomes [2]. The following factors have been suggested to cause anaemia in HF patients: iron deficiency, neurohormonal and proinflammatory activation, renal dysfunction, reduced erythropoiesis, haemodilution, and some medications [2, 3].

The haemodynamic response to tissue hypoxia induced by severe anaemia in patients without cardiac disease presents as tachycardia, high cardiac output induced by the activation of the sympathetic nervous system, low vascular resistance caused by generalised vasodilation, and fluid retention [3–5]. In extreme cases, it can manifest as “noncardiac circulatory congestion” with pulmonary oedema and hypotension [3, 5].

The unfavourable effects of anaemia in HF patients may overlap with pre-existing pathologies. A rise in sympathetic and renin-angiotensin-aldosterone system activity provokes vasoconstriction and reduces renal perfusion. The heart is burdened with an increased volume workload as a result of water and salt retention [4]. Animal studies have revealed that chronic anaemia leads to eccentric cardiac hypertrophy, interstitial fibrosis, increased left ventricular end-diastolic pressure, and decreased systolic functional reserve [5, 6]. Anaemia-related haemodynamic and neurohormonal alterations could potentially result in reduced systolic function related to impaired Frank–Starling responses to preload, particularly in failing hearts [7].

The reaction of the heart to anaemia may differ depending on HF aetiology. Tominaga et al. [4] revealed that, in nonischaemic HF, cardiac output could be increased to deliver sufficient oxygen in response to anaemia, while in ischaemic HF, this standby capacity in the heart seems to be exhausted [4].

The adverse haemodynamic effects of anaemia itself might be frequently missed or underestimated in clinical practice, mostly because of the lack of a simple bedside diagnostic tool. In order to enable timely patient-tailored treatment of acute decompensated HF (ADHF), the evaluation process of harmful haemodynamic effects of anaemia should be simple and fast. Our previous experience with impedance cardiography (ICG) [8, 9], a simple method of noninvasive haemodynamic assessment, encouraged us to verify its usefulness in such clinical settings.

Therefore, the aim of this study was to evaluate haemodynamic profiles of HF patients by means of ICG with respect to the presence of reduced left ventricular ejection fraction (LVEF < 40%) and anaemia.

## 2. Materials and Methods

### 2.1. Study Population

This retrospective secondary analysis included data of 97 patients who were enrolled to the prospective observational study. This study aimed to evaluate the complex pathophysiological background related to HF deterioration with respect to the in-hospital treatment received. The data on the usefulness of noninvasive haemodynamic assessment in admission diagnostics and monitoring of the effects of treatment in patients hospitalised due to ADHF have been previously published [9].

The participants were admitted to the hospital for ADHF (according to the definition of the European Society of Cardiology [1]). We recruited subjects of both sexes aged ≥18 years. Exclusion criteria were as follows: history of acute coronary syndrome within the last 12 weeks, unstable angina, coronary artery bypass grafting surgery within the last 12 weeks, cardiac resynchronisation device implanted within the last 12 months (or planned to be implanted within the next two years), valvular disease/other acquired heart defects requiring surgery, noncardiogenic shock, poorly controlled hypertension, hypertrophic cardiomyopathy, severe chronic obstructive pulmonary disease, bronchial asthma, pulmonary hypertension or other severe lung conditions, pulmonary embolism, acute and/or decompensated noncardiovascular disease, ongoing haemodialysis therapy and/or end-stage chronic kidney disease, severe or chronic infection/inflammatory disease, neoplastic disease, and severe psychiatric disorders. All participants gave their informed consent to be included in this study. This study was registered on ClinicalTrials.gov (NCT02355769) and was approved by the local Bioethics Committee (approval no. 14/WIM/2012).

Clinical examinations covered the history of symptoms, current medication and concomitant diseases by interviews, and review of available medical records. At the time of hospital admission, peripheral venous blood samples were collected for laboratory tests. The certified laboratory analysed the following parameters: creatinine, Hb, urea, haematocrit, high-sensitivity troponin T (hsTnT), N-terminal probrain natriuretic peptide (NT-proBNP), serum iron, total iron-binding capacity, and transferrin saturation. Anaemia was defined as a Hb concentration of <13.0 g/dL in men and <12.0 g/dL in women [1]. The value of the estimated glomerular filtration rate (eGFR) was calculated using the MDRD (Modification of Diet in Renal Disease Study) equation [10].

### 2.2. Echocardiography

Echocardiographic examinations were conducted with the use of Vivid S6/Vivid 7 (GE-Healthcare, USA) ultrasound devices by two experienced echocardiography specialists. We analysed the following parameters: right ventricular end-diastolic diameter (RVEDD, (mm)), left ventricular end-diastolic diameter (LVEDD, (mm)) and left atrial diameter (LAD, (mm)) measured in the parasternal long-axis view, and left ventricular ejection fraction (LVEF, (%)). The median time interval from admission to echocardiography was 3 days.

### 2.3. Impedance Cardiography (ICG)

Impedance cardiography (ICG) is an easy, noninvasive procedure which allows for the assessment of left ventricular (LV) output performance, vascular status, and chest congestion [11]. The impedance measurements were performed within 24 hours of admission (Niccomo™ device, Medis, Germany) by an experienced nurse. Data were recorded during a 10-minute rest assessment in a sitting position and exported to the dedicated software (Niccomo Software). We analysed the following haemodynamic parameters: thoracic fluid content (TFC (1/kOhm)), stroke index (SI (mL/m^2^)) as stroke volume indexed to body surface area, and cardiac index (CI ((mL/min)/m^2^)) calculated as CI = SI × HR (heart rate), and the parameters of left ventricular-aortic outflow are as follows: acceleration index (ACI (1/100 *∗* Ohm/s^2^)), velocity index (VI (1/1000 *∗* Ohm/s)), Heather index (HI (Ohm *∗* s^2^), and systemic vascular resistance index (SVRI ((dyn × s)/cm^5^/m^2^)).

### 2.4. Statistical Analysis

The statistical analysis was performed using Statistica 12.0 (StatSoft, Inc., Tulsa, USA). The distribution and normality of the data were assessed using the Kolmogorov–Smirnov test. Categorical variables were presented as absolute and relative frequencies (percentages), and continuous variables were presented as mean ± standard deviation (SD). The study group was stratified by the presence of reduced LVEF (<40% vs ≥40%) and anaemia (anaemic vs nonanaemic). The subgroups were labelled as follows: subgroup A (anaemic, LVEF < 40%), subgroup B (nonanaemic, LVEF < 40%)), subgroup C (anaemic, LVEF ≥ 40%), and subgroup D (nonanaemic, LVEF ≥ 40%). They were compared in terms of clinical, echocardiographic, and haemodynamic parameters with the use of ANOVA/Kruskal–Wallis test for continuous variables and chi-squared or Fisher's exact test for categorical variables. A *p* value of <0.05 was considered statistically significant.

## 3. Results and Discussion

### 3.1. Basic Characteristics

The study group (mean age 71.5 ± 12.6 years) was predominantly male (*n* = 77; 79.4%; Table 1). The mean LVEF was 37.3 ± 14.1%, and 59 (60.8%) subjects presented with LVEF < 40%. Many subjects (*n* = 62/63.9%) reported symptoms of NYHA (New York Heart Association) class III and approximately one-third (*n* = 35/36.1%) had resting dyspnoea (NYHA class IV). The most common comorbidities were arterial hypertension, atrial fibrillation, and diabetes (Table 1). The mean concentration of haemoglobin was 12.6 ± 2.3 g/dL, and anaemia was confirmed in 52 patients (53.6%).

### 3.2. Comparison between Subgroups for Basic Clinical Characteristics

**Figure 1:**
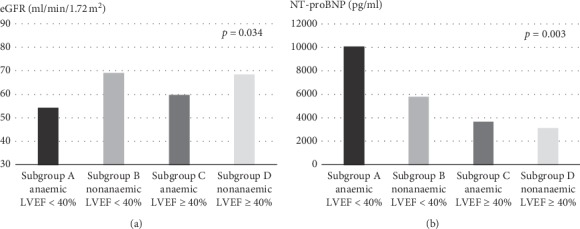
Comparison of mean values of eGFR (estimated glomerular filtration rate) and NT-proBNP (N-terminal probrain natriuretic peptide).

As compared with those with LVEF < 40% (subgroups A/B), subgroups with LVEF ≥ 40% (subgroups C/D) comprised older patients, with a relatively higher percentage of women who presented with arterial hypertension and lower NT-proBNP concentrations (Table 2; Figure 1). At the same time, anaemic subgroups (subgroups A/C vs. B/D) were distinguished by higher prevalence of a previous history of atrial fibrillation and chronic kidney disease, higher concentration of creatinine and lower eGFR, and a tendency to have lower iron and transferrin saturation (Figure 1). A significantly higher NT-proBNP in anaemic patients with low LVEF was particularly distinctive (subgroup A) (Figure 1). No significant intersubgroup differences in functional NYHA class were observed.

### 3.3. Comparison between Subgroups for Echocardiography and Impedance Cardiography

Patients with significantly impaired systolic function (subgroups A/B) and those with anaemia and LVEF ≥ 40% (group C) were characterised by a more greatly extended left atrium in comparison with nonanaemics with LVEF ≥ 40% (subgroup D). Only the LVEF criterium was of significance with respect to the LV dimension (Table 3, echocardiography). There was also a correlation between impaired LVEF (subgroups A/B) and lower BP (Table 3, impedance cardiography). However, the presence of anaemia was not correlated with reduced LVEF (subgroups A vs. B and C vs. D).

**Figure 2:**
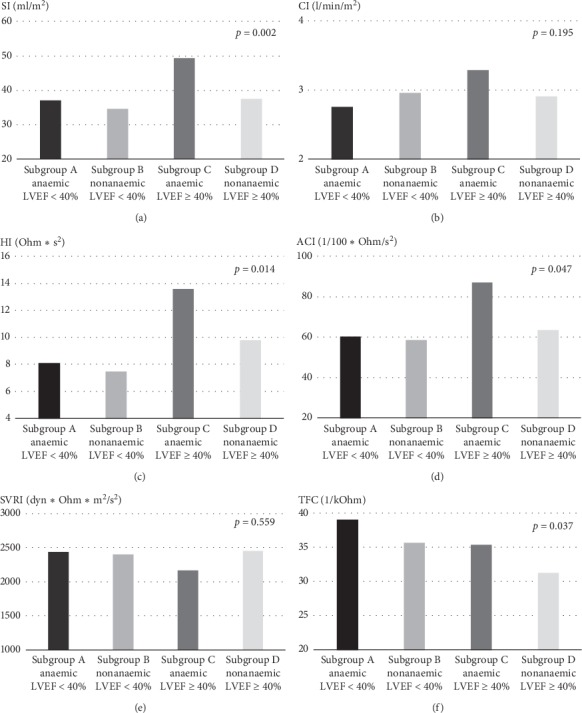
Subgroup comparison of mean values of haemodynamic parameters measured by means of ICG (ACI, acceleration time index; CI, cardiac index; HI, Heather index; SI, stroke index; SVRI, systemic vascular resistance index; TFC, thoracic fluid content).

Impedance cardiography revealed that anaemic patients without reduced LVEF (subgroup C) were distinguished by significantly higher markers of LV pumping performance (SI, HI, and ACI) (Table 3, impedance cardiography; Figure 2). In addition, anaemic patients (subgroups A and C) presented with higher TFC than nonanaemic patients (subgroups B and D).

### 3.4. Discussion

The results of impedance cardiography suggest that the haemodynamic response to anaemia in HF patients is related to the severity of LV impairment. This simple and noninvasive diagnostic method complements traditional diagnostics. Impedance cardiography confirmed the presence of anaemia-related water retention despite LVEF, whereas increased LV performance (SI, HI, ACI) was observed only in subjects with LVEF ≥ 40%. However, the fact that NT-proBNP and NYHA class intersubgroup comparison did not reveal any clinical benefit of this phenomenon is of clinical importance. Moreover, hearts with significantly decreased LVEF seemed to be unable to respond effectively to anaemia-related compensatory mechanisms, consequently leading to an increased LV load and chest congestion.

Anaemia is considered to be an independent risk factor for cardiovascular adverse outcomes, both in the general population and in cardiovascular patients [12–14]. Under normal conditions, reduced tissue oxygenation due to chronic anaemia results in compensatory responses to enhance oxygen-carrying capacity. The anaemia-mediated high-output state initially supports an increase in oxygen transport, but in the long term, it contributes to the worsening of cardiovascular function, especially in HF patients [2, 15].

This effect can be observed even at the asymptomatic stage of cardiovascular dysfunction. Zhou et al. [15] observed that non-HF subjects with severe anaemia (6–9 g/dL) presented with LV enlargement, LV hypertrophy, and impaired systolic function assessed by means of 3-dimensional speckle-tracking echocardiography (3DSTE). This proved that a persistently hyperdynamic circulatory state associated with chronic volume overload resulted in increased LV filling pressure, LV remodelling, and systolic dysfunction.

Anaemia triggers several unfavourable effects. Vasodilation-related low blood pressure may stimulate neurohormonal activation. Increased sympathetic stimulation reduces renal blood flow and the glomerular filtration rate. Moreover, it contributes to the activation of the renin-angiotensin-aldosterone axis, nonosmotic release of vasopressin, as well as salt and water retention. Consequently, this leads to an increase in total body water, extracellular volume, and plasma volume [3, 16].

In our cohort, the haemodynamic effect of anaemia was strongly demonstrated in subjects with mildly impaired LV systolic function (subgroup C). Relatively higher values of ICG-derived parameters characterising LV output performance (SI, CI, HI, ACI, and VI) were accompanied by lower vascular tone (SVRI) and higher chest fluid accumulation (TFC). An increase in left atrium dimension appeared to complement the pattern of “high output, high volume, high left chamber pressure, and pulmonary congestion.” ICG proved to be useful in the haemodynamic profiling of HF patients. In our previous study conducted on this cohort, we proved its usefulness in identifying the differences between patients with significantly impaired LV systolic function versus those with mildly impaired and preserved LV systolic function [17].

Natriuretic peptides are cardiac neurohormones released by the ventricles in response to ventricular wall tension and stretch [1]. Therefore, one can conclude that distinctively high NT-proBNP concentrations in subgroup A (anaemic subjects with reduced LVEF) seem to confirm the detrimental effect of anaemia-related volume overload on a damaged myocardium. In combination with the presence of high TFC and no “high-output effect,” this suggests that failing hearts operating on the plateau of the Frank–Starling curve are unable to appropriately increase their performance.

There are reports revealing the prognostic significance of anaemia in HF patients. In patients from the Swedish HF Registry (*n* = 49,985), anaemia revealed to be related with increased risk of mortality or HF hospitalisations (composite endpoint), greater in patients with less impaired LVEF (HR for preserved ≥50% and midrange (40–49%) vs. reduced (<40%): 1.24 and 1.26 vs 1.14; *p*_interaction_ = 0.003) [18]. Ralli et al. [19] conducted a study on a cohort of 264 patients with advanced HF (mean LVEF 24%), showing that low Hb concentration in the setting of elevated B-type natriuretic peptide (BNP) is associated with markedly increased mortality. Patients without anaemia and with lower BNP levels had an excellent prognosis with a 96.3% one-year survival rate. In contrast, only 64.7% of those with anaemia and elevated BNP (*p* < 0.001) survived, representing a 10.4-fold increased risk of death. Listerman et al. [20] evaluated 209 HF patients with an ejection fraction of ≤40% who underwent haemodynamic exercise testing and reported that the resting right atrial pressure was higher (10 mm Hg vs. 8 mm Hg; *p*=0.02) and the exercise peak VO_2_ was significantly lower (11.7 mL/min/kg vs. 13.4 mL/min/kg; *p*=0.01) among the anaemic patients. No significant differences in parameters characterising cardiac output (stroke volume and cardiac index) at peak exercise were observed between anaemic and nonanaemic patients. This suggests that the desired compensatory haemodynamic response to anaemia may be limited in patients with significantly reduced LVEF.

Anaemic patients are expected to have a more pronounced haemodynamic response to exercise with respect to increased cardiac output. However, exercise haemodynamic adaptation to anaemia in HF patients is insufficient and may partially explain poorer exercise tolerance. Horwich et al. [21] observed that patients with lower Hb are more likely to present with a NYHA functional class of IV, lower peak VO_2_, and reduced survival rate (an increase in relative risk of death of 1.13 per 1 g/dL decrease in Hb). Moreover, Wang et al. [22] reported that anaemia limited the beneficial effects of aerobic interval training in anaemic HF subjects; nonanaemic HF subjects conversely achieved slightly better results in terms of peak VO_2_ and O_2_ uptake efficiency slope, as well as cerebral flow, during exercise. This may partly explain why subgroup C of our cohort failed to achieve better functional capacity (NYHA class) than the other subgroups, despite presenting with a resting high-output state.

It is also worth mentioning that our results confirmed the previously reported strong correlation between anaemia and comorbidities of negative prognostic value [1–3, 23]. Patients with decreased Hb had a higher prevalence atrial fibrillation and chronic kidney disease in medical history, as well as higher creatinine concentration and lower eGFR. They also tended to have lower serum iron concentrations and lower transferrin saturation.

### 3.5. Clinical Implications

Our results show that the haemodynamic effects of anaemia may differ individually among HF patients. The results of ICG suggest that anaemia-related “high cardiac output” state is related to milder cardiac impairment. However, this is not tantamount to clinical benefit because this state is rather characterised as “high intravascular volume” than “high cardiac contractility” and should be targeted by individualised therapy. The harmful effect of anaemia in patients with low LVEF seems to be clearly depicted by high NT-proBNP and TFC. These results should be interpreted with consciousness that HF, as a complex syndrome, may involve other organs influencing haemoglobin level such as the lungs, kidney, liver, and haematopoietic system [1]. The patient's prognosis is the result of all these abnormalities. The complex relation between HF and anaemia supports the need for individualised haemodynamic profiling that seems to be easily satisfied in emergency settings with the use of ICG.

Our results support recommendation to carefully investigate the causes of anaemia in HF patients, especially the potentially reversible ones (e.g., iron deficiency, chronic bleeding, and medications) [1, 24]. It is suggested that the treatment for anaemia should be initiated at an early stage before systolic function begins to decline [4]. Intravenous iron infusions seem to be the most efficient therapy for anaemia in HF. Correction of iron deficiency enhances physical exercise capacity and the quality of life, while reducing symptoms and the number of unscheduled hospital admissions [25]. In hypervolemic patients, it is also worth excluding haemodilution-related anaemia, especially in advanced HF. Prospective trials evaluating the effects of anaemia treatment in ADHF are still needed, and the haemodynamic response to such a therapeutic strategy should be further investigated.

### 3.6. Limitations

The main limitation of our study was the small size of the evaluated subgroups, which undoubtedly affected the statistical power of the analysis. For this reason, the patients with non-reduced LVEF were combined into one subgroup. We also did not use cardiac magnetic resonance imaging that might provide additional data about heart morphology and function. Because of the small sample size, the analysis was underpowered for multivariate models, including potential confounders such as sex and age. We also did not differentiate in detail ADHF from end-stage HF patients that should be considered in future studies aimed to assess the prognosis. Another important limitation was the 24-hour window in which ICG assessment was performed because haemodynamic profiles can change less than an hour after initiating effective treatment. We would also like to emphasise the fact that only the fluid content within the thorax can be measured by ICG, and this significantly restricts any possible conclusions regarding the overall body fluid content. We also did not perform exercise evaluation, and this limits our ability to comment on the effect of anaemia on exercise capacity and haemodynamics. The effect of potential haemodilution should also be taken into consideration in patients with ADHF and low Hb [26].

## 4. Conclusions

In ADHF, anaemia is related with fluid overload, relatively higher cardiac systolic performance but no clinical benefit in patients with preserved/midrange LVEF, and increased left ventricular tension, fluid overload, and impaired cardiac systolic performance in patients with reduced LVEF. Concomitant fluid overload and impaired renal function are suggestive of a poorer prognosis.

## Figures and Tables

**Table 1:** Basic characteristics of the study group.

	Study group *n* = 97
Age (years)	71.5 ± 12.6
Men	77 (79.4)
NYHA class: III/IV	62 (63.9)/35 (36.1)
Atrial fibrillation	61 (62.9)
Arterial hypertension	64 (66.0)
Chronic obstructive pulmonary disease	15 (15.5)
Chronic kidney disease	28 (28.9)
Diabetes mellitus	48 (49.5)
Creatinine (mg/dl)	1.31 ± 0.51
eGFR (ml/min/1.72 m^2^)	62.2 ± 23.6
Urea (mg/dl)	54.2 ± 26.6
hsTnT (ng/L)	106.9 ± 263.6
NT-proBNP (pg/ml)	6214 ± 7196
Haemoglobin (g/dl)	12.6 ± 2.3
LVEF (%)	37.3 ± 14.1
LVEF category: <40%/40–49%/≥50%	59 (60.8)/12 (12.4)/26 (26.8)
Pharmacotherapy at admission	
Beta-blockers	74 (76.3)
Angiotensin-converting-enzyme inhibitors/angiotensin receptor blockers	58 (59.8)/10 (10.3)
Mineralocorticoid receptor antagonist	31 (32.0)
Diuretic	69 (71.1)

Data are presented as mean ± SD or *n* (%). eGFR, estimated glomerular filtration rate; LVEF, left ventricular ejection fraction; NYHA, New York Heart Association; NT-proBNP, N-terminal probrain natriuretic peptide; hsTnT, high-sensitivity troponin T.

**Table 2:** Comparison between subgroups for basic clinical characteristics.

		Subgroup A anaemic, LVEF < 40% *n* = 30	Subgroup B nonanaemic, LVEF < 40% *n* = 29	Subgroup C anaemic, LVEF ≥ 40% *n* = 22	Subgroup D nonanaemic, LVEF ≥ 40% *n* = 16	*p*
Anamnesis and physical examination						
Age (years)		73.3 ± 9.8	62.7 ± 14.2	77.7 ± 8.3	75.3 ± 11.1	0.00003
						B vs A^#^
						B vs C^#^
						B vs D^#^
Men		26 (86.7)	24 (82.8)	18 (81.8)	9 (47.5)	0.001
NYHA class: III/IV		15 (50.0)/15 (50.0)	22 (75.9)/7 (24.1)	14 (63.6)/8 (36.4)	11 (57.9)/5 (42.1)	0.469
Atrial fibrillation		18 (60.0)	11 (37.9)	16 (72.7)	6 (37.5)	0.042
Arterial hypertension		15 (50.0)	19 (65.5)	16 (72.2)	14 (87.5)	0.067
Chronic obstructive pulmonary disease		5 (16.7)	5 (17.2)	3 (12.6)	2 (12.5)	0.966
Chronic kidney disease		13 (44.8)	3 (10.3)	9 (40.9)	3 (18.8)	0.013
Diabetes mellitus		15 (50.0)	14 (48.3)	16 (72.2)	3 (18.8)	0.013

Laboratory tests						
Creatinine (mg/dl)		1.50 ± 0.50	1.21 ± 0.44	1.40 ± 0.66	1.03 ± 0.24	0.012
						A vs D^*∗*^
eGFR (ml/min/1.72 m^2^)		54.4 ± 23.1	69.2 ± 23.3	59.8 ± 25.4	68.6 ± 18.1	0.034
Urea (mg/dl)		59.6 ± 24.7	51.9 ± 33.7	58.7 ± 24.6	41.9 ± 11.3	0.137
hsTnT (ng/L)		134.0 ± 269.3	114.4 ± 319.5	54.8 ± 64.4	111.3 ± 317.2	0.767
NT-proBNP (pg/ml)		10105 ± 8825	5806 ± 7613	3666 ± 3083	3159 ± 3033	0.003
						A vs B^0.075^
						A vs C^*∗∗*^
						A vs D^*∗∗*^
Haemoglobin (g/dl)		11.6 ± 1.3	14.7 ± 1.3	10.3 ± 1.7	13.7 ± 1.8	<0.000001
Haematocrit (%)		35.4 ± 3.4	44.3 ± 3.7	32.5 ± 3.4	41.7 ± 6.2	<0.000001
Iron (*μ*g/L)		47.4 ± 20.9	67.0 ± 34.3	53.5 ± 25.7	62.9 ± 31.8	0.078
Transferrin saturation (%) data of 60 subjects		15.4 ± 7.1	20.8 ± 14.5	18.5 ± 11.5	20.2 ± 10.5	0.297

Data are presented as mean ± SD or *n* (%); ^*∗*^*p* < 0.05; ^*∗∗*^*p* < 0.01; ^#^*p* < 0.001. eGFR, estimated glomerular filtration rate; NYHA, New York Heart Association; NT-proBNP, N-terminal probrain natriuretic peptide; hsTnT, high-sensitivity troponin T.

**Table 3:** Comparison between subgroups for echocardiography and impedance cardiography.

	Subgroup A anaemic, LVEF < 40% *n* = 30	Subgroup B nonanaemic, LVEF < 40% *n* = 29	Subgroup C anaemic, LVEF ≥ 40% *n* = 22	Subgroup D nonanaemic, LVEF ≥ 40% *n* = 16	*p*
Echocardiography					
LVEDD (mm)	65.6 ± 8.1	64.4 ± 8.8	52.7 ± 6.6	49.6 ± 6.4	<0.00001
					A vs C^#^
					A vs D^#^
					B Vs C^#^
					B Vs D^#^
RVEDD (mm)	36.0 ± 5.9	36.7 ± 6.6	35.1 ± 4.5	31.7 ± 4.1	0.070
LAD (mm)	49.1 ± 5.4	48.3 ± 5.0	47.8 ± 6.3	42.7 ± 6.2	0.007
					A vs D^*∗∗*^
					B Vs D^*∗*^
					3 vs D^*∗*^
LVEF (%)	27.6 ± 6.7	27.7 ± 6.3	51.3 ± 8.5	53.4 ± 8.2	<0.00001
					A vs C^#^
					A vs D^#^
					B Vs C^#^
					B Vs D^#^

Impedance cardiography (haemodynamics)					
HR (bpm)	76.0 ± 18.4	91.9 ± 24.3	73.9 ± 16.6	82.9 ± 23.9	0.060
SBP (mmHg)	112.4 ± 16.9	116.9 ± 16.8	133.0 ± 30.8	140.4 ± 28.2	0.006
					A vs C^*∗∗*^
					A vs D^#^
DBP (mmHg)	71.1 ± 8.3	76.4 ± 13.8	69.0 ± 11.9	76.6 ± 11.0	0.047
SI (ml/m^2^)	37.2 ± 11.2	34.7 ± 9.6	49.4 ± 14.3	37.6 ± 9.7	0.002
					A vs C^*∗∗*^
					B Vs C^#^
					D vs C^*∗*^
CI (l/min/m^2^)	2.76 ± 0.88	2.96 ± 0.68	3.29 ± 0.92	2.91 ± 0.59	0.195
HI (Ohm *∗* s^2^)	8.1 ± 5.0	7.5 ± 4.6	13.6 ± 7.6	9.8 ± 4.8	0.014
					A vs C^*∗∗*^
					B Vs C^*∗∗*^
ACI (1/100 *∗* Ohm/s^2^)	60.4 ± 25.2	58.8 ± 21.7	87.2 ± 40.4	63.6 ± 34.0	0.047
					A vs C^*∗*^
					B Vs C^*∗*^
VI (1/1000 *∗* Ohm/s)	39.4 ± 15.6	36.9 ± 16.1	55.7 ± 28.4	38.0 ± 17.1	0.098
SVRI (dyn *∗* s *∗* m^2^/cm^5^)	2442 ± 765	2405 ± 713	2168 ± 857	2451 ± 726	0.559
TFC (1/kOhm)	39.1 ± 7.7	35.7 ± 8.5	35.4 ± 7.1	31.3 ± 4.8	0.037
					A vs D^*∗*^

Data are presented as mean ± SD/*n* (%); ^*∗*^*p* < 0.05; ^*∗∗*^*p* < 0.01; ^#^*p* < 0.001. ACI, acceleration time index; CI, cardiac index; DBP, diastolic blood pressure; HI, Heather index; HR, heart rate; LAD, left atrium diameter; LVEDD, left ventricular end-diastolic diameter; LVEF, left ventricular ejection fraction; RVEDD, right ventricular end-diastolic diameter; SBP, systolic blood pressure; SI, stroke index; SVRI, systemic vascular resistance index; TFC, thoracic fluid content; VI, velocity index.

## Data Availability

The data used to support the findings of this study are available from the corresponding author upon request.
